# Prevalence of hypertension and associated factors in Jalalabad City, Nangarhar Province, Afghanistan

**DOI:** 10.5195/cajgh.2015.134

**Published:** 2015-02-05

**Authors:** Khwaja Mir Islam Saeed

**Affiliations:** Afghanistan National Public Health Institute, Ministry of Public Health, Kabul, Afghanistan

**Keywords:** prevalence, associated factors, hypertension, urban, Afghanistan

## Abstract

**Background:**

Hypertension affects an estimated one billion people, worldwide. It is a public health challenge characterized by increased morbidity, mortality, as well as cost to the community and health systems. The goal of this study is to determine the prevalence of hypertension and characterize associated risk factors in an urban setting in Afghanistan.

**Methods:**

A cross-sectional study of adults aged 25-65 years was conducted in Jalalabad city from May to June 2013 using the World Health Organization STEPwise approach to surveillance (WHO STEPS). A multistage technique was used to enroll 1,200 participants in the study. Demographic and socio-economic variables were collected via individual interviews using the WHO STEPS survey, after which blood samples were collected using a locally developed standard operating procedure (SOP). Bivariate and multivariable analyses were performed to explore the association between hypertension and associated factors.

**Results:**

A total of 1,180 adults (40% males, 60% females) of 25–65 years of age were surveyed. The response rate was 98.5 % and the prevalence of hypertension was 28.4. Independent risk factors of hypertension were found to be: age ≥ 50 (AOR = 3.42, 95% CI: 2.50 – 4.76); sex (AOR = 0.58, 95% CI: 0.38 – 0.88); obesity (AOR = 2.1, 95% CI 1.49 – 2.94); and diabetes (AOR = 1.75, 95% CI: 1.10 – 2.79). Independent protective factors were physically demanding occupations (AOR = 0.55, 95% CI: 0.36 – 0.85); physical activity itself (AOR = 0.69, 95% CI: 0.47 – 0.99) and consuming more vegetables (AOR = 0.59, 95% CI: 0.38 – 0.93).

**Conclusion:**

This urban setting in Afghanistan evidenced a high prevalence of hypertension; age, obesity, and diabetes were identified as risk factors and physical activity and consuming more vegetables were protective. These findings have implications for future public health intervention and clinical efforts.

Hypertension (HTN) is a global public health problem, affecting approximately one billion people worldwide, a figure that is predicted to increase to 1.5 billion by the year 2025.^1^ The global prevalence of HTN is approximately 30% among adults; in developed countries, prevalence is beginning to stabilize or decrease, while in the developing regions, proportions continue to rise (between 20–50%).^2^,^3^ HTN is a global problem with some of the commen risk factors reported in the litearature being genetics, family history, advanced age, race, obesity, physical inactivity, lifestyle, cigarette smoking, excessive salt and alcohol intake, and dietary habits.^4^–^7^ HTN prevalence has been reported to be 15–35% in Asia,^8^ 20–33% in Africa,^9^ 18–22% in the USA,^10^ 44% in some European countries,^10^ 44% in Turkey,^4^ 26.3% in Egypt,^11^ 32.2% in India,^12^ and 32.1% in Qatar.^13^ In the Eastern Mediterranean Region (EMR), the prevalence of HTN has been estimated to be 29%, affecting approximately 125 million individuals.^14^

In Afghanistan, due to years of war and conflict, few studies have been conducted to estimate the burden of hypertension. According to our previously published study of chronic disease risk factors in Kabul, in a sample of individuals aged 40+, the overall prevalence of obesity, HTN, and diabetes mellitus was 31.2%, 46%, and 13.3%, respectively.^15^ However in neighboring countries such as Iran, the overall prevalence of HTN in the adult population was 23%, with 50% of those aged >55 years affected.^16^ Previously published research from Pakistan reported an overall prevalence of HTN of 26% which diffiered by 34% among males and 24% in females.^17^

According to anecdotal reports from clinicians in Kabul, the number of people with HTN is increasing. In addition, the recent Afghan Mortality Survey (AMS 2010) survey indicated that 35% of all-cause mortality in Afghanistan is due to non-communicable disease, particularly cardiovascular disease and cancer.^18^ As of 2014, Afghanistan suffers from lack of reliable information on the burden of non-communicable disease, including HTN, due to the fact that high priority is given to the investigation of infectious diseases. The purpose of this study is to estimate the burden of HTN and associated risk factors among the adult population in Afghanistan’s eastern city, Jalalabad. This information is essential to provide evidence to support strategic decisions such as resource allocation and public health interventions to reduce risk factors and decrease the burden of disease.

## Methods and Materials

We conducted a cross-sectional study using the World Health Organization STEPwise approach to surveillance (WHO STEPS)^19^ to estimate the prevalence and factors for non-communicable diseases in Jalalabad city, Afghanistan. STEPS was initiated by the WHO to establish the surveillance of risk factors for noncommunicable diseases. The survey tool collects information on behavioral, physical and biochemical measurements as a part of the core, expanded, and optional modules.^19^ Each country can use and modify the modules and steps based on their needs.

### Setting

Jalalabad is a city in the Nangarhar province on the eastern border of Afghanistan. Five districts were selected for assessment while one was excluded due to safety concerns. Inclusion criteria included: ages 25–64 (adult population, as outlined in WHO survey tool), city residents during study period, and consent to participate. Exclusion criteria included: refusal to participate and temporary residency. In addition, all five districts were classified into clusters, sub-clusters, target areas, and then households. In the series of households, every third household and one eligible adult in each household was selected for research assessment. The response rate was 98.5% with a 1.5% refusal to participate. Temporary residents (less than six months) and those living in institutionalized settings along with unsafe areas were excluded from the survey. We excluded temporary residents because our goal was to obtain a reliable data about permanent residents, not migrant populations.

### Population

Over 60% of the participants were female. Thus, we adjusted for sex in our analyses. After informing the community representatives, we approached all four clusters (A, B, C, D) and 20 sub-clusters of EPI, including five city districts, to obtain the target population. Our primary sampling unit (PSU) was sub-clusters, secondary sampling units (SSU) were streets/areas, tertiary sampling units (TSU) were households, and ultimate sampling units (USU) were respondents more than 25 years of age in the household. The interviewer was instructed to find a the famous masjid as a fixed landmark or a very populated street within the boundaries of the selected location and following the bottle rotating rule to proceed to series of households. At last level random selection was carried out by writing the name of target members on a separate piece of paper and then drawing the names.

### Measures

As mentioned above, the WHO STEPS was used to collect demographic, socio-economic, clinical, and behavioral data via face-to-face interviews. Weighing scales and tension tape were used to measure body weight and height. A body mass index (BMI, hereafter reported without units) ≥ 30 kg/m^2^ were considered as obese, 25–29.9 was considered as overweight, and 18.5–24.9 was considered normal weight.^20^ A waist circumference ≥ 94 cm for men and ≥80 cm for women was considered as central obesity.^21^ Cuff type sphygmomanometers were used to measure systolic and diastolic blood pressure twice with five minutes between each measurement at a sitting or lying position by our trained surveyors. Systolic blood pressure levels ≥ 140 mmHg and diastolic pressure levels ≥ 90 mmHg were considered hypertensive.^22^ HTN in this study was defined as having a previous diagnosis of HTN or having a BP of HTN status. Blood samples were collected and processed by lab technicians under supervision of lab coordinator. After shipment of samples to the Central Public Health Laboratory (CPHL) in Kabul, they were stored at −80°C until glucose measurement was completed. Close monitoring of all study steps by core group of investigator was implemented to enhance quality of data at all research stages. The study protocol was approved by institutional review board (IRB) at the ministry of public health, Afghanistan. Informed consent was provided by all study participants.

### Statistical Considerations

Data entry was performed using Epi Info version 3.5.1.^23^ Analyses were performed using IBM SPSS software version 20.^24^ As data regarding risk factor prevalence in this province were not available, we assumed the highest prevalence and 95% confidence interval and band of error of 5%. To balance considerations of non-response rate, cost, resources, and time without compromising the representativeness of the sample, a two-phase cluster sampling technique was used. The sample size was calculated to be able to determine the effect of risk factors on non-communicable diseases. The resulting sample size was 1,200. Data were collected May–June 2013. Participants with missing data for blood pressure were excluded from the final analysis, which incorporated 1,180 participants. Pregnant women were also excluded from obesity-related analyses. Logistic regression was used to examine the association of relevant variables adusting for all other vaiables and to calculate the adjusted odds ratio (AOR).

## Results

### Descriptive Analysis:

The average age this sample was 39.16 ± 11.5 years. The overall prevalence of HTN was 28.4% among age group of 25–65 years. Mean systolic blood pressure and standard deviation (SD) was 122 ± 20 mmHg and ranged from 70 to 220 mmHg. Mean diastolic blood pressure was 79 ± 13 mmHg and ranged from 40 to 130 mmHg.

Overall, 4% of hypertensive participants were previously diagnosed or were under treatment for HTN, which is very low and could be due to latent HTN or asymptomatic HTN. More than half (66.9%) were illiterate and 66.2% had income of less than 10,000 AFN (200USD) per month (Refer to Table 1 for details on demographic variables).

The mean height, weight and waist circumference were 161.4 cm, 69.3 Kg and 85.4 cm respectively. The mean and SD of body mass index was 26.8 ± 6.8 Kg/m^2^. Descriptive statistics demonstrated that 7.6% of respondents were current smoker and on average they smoked 12 cigarettes daily. 13.3% were mouth snuff users which are almost double of cigarettes smoker. Around 54% of study participants were using solid oil in their kitchen for cooking. As we had data on number of days per week that research participants consumed fruit (average number of fruit servings per day), these data were categorized dichotomous by cut of three days per week. Data shows 65% of participants were consuming fresh fruits less than three days per week as compared to 27% who were consuming fruits more than three days per week. These variables have been described in Table 2.

The study demonstrates that 32% of respondents were employed at jobs that required high level of physical activity and 49% moderate physical activity. Farmers, workers and business were categorized as high physical work and the office related jobs categorized low physical group. The proportions of pathophysiological factors potentially associated with HTN were diabetes (11%), overweight (30%), obesity (23%), and central obesity (50%). The biochemical measurements findings shows the mean and SD total triglycerides, cholesterol, HDL, LDL and fasting blood sugar were 187.5 ± 76.5, 198.5 ± 42, 39.16 ± 8, 122.3 ± 41.6, and 92.3 ± 39.5 mg/DL, respectively.

### Inferential Analysis:

According to bivariate analysis, hypertensive status increased incrementally with age with highest prevalence in age group of 45–55 years old (Table 3).

There was a significant association between HTN and sex; females were two times more likely to be affected by HTN than males. In terms of education status, those who were illiterate were 1.3 times (95% CI: 1.01 – 1.76) at greater risk for developing HTN. We found significant associations between the level of income and proxies of physical activities with hypertension. Smoking habits, mouth snuff use and diet were associated with HTN but it was not statistically significant. Overweight and obesity were significantly associated with HTN (overweight OR = 2.52, 95% CI: 1.28 – 4.98), (obesity OR = 4.55, 95% CI: 2.30 – 8.99). Those who were HTN had 2.32 (95% CI: 1.61 – 3.36) times higher odds of being diabetic compared with normal blood pressure. We did not find any significant relationship between level of blood lipids and HTN (Table 4).

Multivariate analysis (Table 5) demonstrated that older age (AOR = 3.42, 95% CI: 2.50 – 4.76), sex (AOR = 0.58, 95% CI: 0.38 – 0.88), physically demanding jobs (AOR = 0.55, 95% CI: 0.36 – 0.85), general obesity (AOR = 2.1, 95% CI: 2.11 – 2.94), diabetic status (AOR = 1.75, 95% CI: 1.10 – 2.97), physical activity (AOR = 0.69, 95% CI: 0.47 – 0.99), and consuming more vegetables (AOR = 0.38, 95% CI: 0.38 – 0.93) were independently associated with HTN. Sex was not associated with HTN.

## Discussion

This is one of the few published studies on HTN prevalence in Afghanistan. Our findings suggest that HTN, which affected one third of this sample, is a growing public health challenge in the urban setting of Jalalabad. However, comparing with other regions, it was lower than in Kabul, the capital of the country.^15^–^17^

Almost 60% of the study participants were female compare to 49% in general population. The main reason for this difference was employment of men outside the house during day time and not available for study assessment. Females were at higher risk of HTN, a finding that other investigations support.^17^,^25^ This disparity may be explained by females engaging in less physical activities at home or evidencing higher proportions of obesity; however additional studies are needed to test this hypothesis. Health care systems should be strengthened so they have the capacity for early detection and the means for effective treatment of those affected with HTN.

Results shows 7.6% of respondents were smoking currently daily while double of that were mouth snuff users. It could be due to low cost of snuff as compare to cigarettes. The saturated (solid) ghee for kitchen considered a risk factor for HTN and obesity while the unsaturated (liquid) ghee was considered to have lower risk. Physical activity and diet rich in vegetables were protective factors against HTN in this study. These findings have been corroborated by other authors exploring hypertensive disease in various parts of the world.^13^,^26^ In addition, obesity and diabetes were found to be the independent risk factors for HTN in this study, which is supported by previously published studies.^27^–^29^

Based on this study, a baseline understanding of HTN prevalence in the region could be established for Jalalabad city, while further studies and public health programs can be established to compare rates in other geographic settings and evaluate interventions. Screening individuals over the age of 40, particularly for females, is recommended based on findings of this study for urban settings of Afghanistan. Policy changes are essential to reduce risk of HTN in various populations within Afghanistan, including public education to improve dietary habits and enhance physical activity. Furthermore, prevention strategies should focus on risk factors for metabolic syndrome, such as obesity and diabetes. Due to the government’s focus on communicable diseases, lesser emphasis is given to non-communicable disease such as HTN.^30^ Recently, the national strategy for non-communicable diseases has been finalized in the country.^31^ Therefore, prevention and control of HTN needs political will, combined with community support and behavioral change on the part of the individuals and their families.

## Figures and Tables

**Table 1:** Participant characteristics among those surveyed using the WHO STEPS in Jalalabad, Nangarhar, Afghanistan

**Variables**	**Categories**	**Un-weighted**		**Weighted**	

		**N**	**%**	**N**	**%**
**Age**					
	25 – 34	445	37.7	450	38.2
	35 – 44	305	25.8	281	23.9
	45 – 54	207	17.5	211	17.9
	54 and over	128	10.8	144	12.3
	Missing	95	8.1	91	7.7
**Sex**					
	Female	579	49.1	715	60.6
	Male	600	50.9	465	39.4
**Level of education**					
	Illiterate	841	71.3	779	66.9
	Primary/Unofficial Education	140	11.9	163	13.8
	Secondary School	135	11.4	158	13.4
	University and more	53	4.5	65	5.5
	Missing	11	0.9	13	1.1
**Residence**					
	District 1	176	14.9	196	16.7
	District 2	163	13.8	138	11.8
	District 3	299	25.3	275	23.3
	District 4	302	25.6	354	30.1
	District 5	240	20.3	214	18.1
**Work status**					
	Official Employee	109	9.2	131	11.2
	Business	78	6.6	101	8.6
	Farmer/worker	222	18.8	287	24.5
	Homemaker	632	53.6	506	43.2
	Unable to work/retired	80	6.8	102	8.7
	Refused	54	4.6	45	3.9
	Missing	5	0.4	5	0.4
**Monthly income (Afghanis)**					
	≤ 10,000	698	59.2	736	62.5
	10,000 – 20,000	41	3.5	42	3.6
	≥ 20,000	42	3.6	34	2.9
	Refused	398	33.7	363	30.9
	Missing	1	0.1	1	0.1
**Marital status**					
	Single	88	7.5	95	8.1
	Married	1,039	88.1	1,036	88
	Widow/widower	44	3.7	38	3.2
	Refused	9	0.8	8	0.7

**Table 2:** Frequency distribution of behavior risk factors of those surveyed using the WHO STEPS in Jalalabad, Nangarhar, Afghanistan

**Variables**	**Categories**	**Female (%)**	**Male (%)**	**Total (%)**
Smoking status*				
	No	535 (51.3)	507 (48.7)	1,042 (92%)
	Yes	1 (1.1)	89 (98.9)	92 (8.0)
**Smoking duration in years**				
	< 10 years	0 (0.0)	58 (100.0)	58 (43.3)
	10 – 20 years	3 (6.1)	46 (93.9)	49 (36.6)
	> 20 years	0 (0.0)	27 (100.0)	27 (20.1)
Mouth snuff use*				
	No	536 (54.6)	446 (45.4)	982 (86.2)
	Yes	3 (1.9)	154 (98.1)	157 (13.8)
Fruit servings consumed in days per week*				
	≤ 3 days	399 (52.2)	366 (47.8)	765 (70.2)
	> 3 days	143 (44.1)	181 (55.9)	324 (29.8)
Vegetable servings consumed in days per week*				
	≤ 3 days	150 (65.2)	80 (34.8)	230 (19.7)
	> 3 days	418 (44.7)	517 (55.3)	935 (80.3)
Type of kitchen oil used*				
	Liquid	365 (77.8)	104 (22.2)	469 (42.1)
	Solid	170 (26.4)	475 (73.6)	645 (57.9)
Vigorous physical activity*				
	No	305 (40.6)	446 (59.4)	751 (66.8)
	Yes	223 (59.6)	151 (40.4)	374 (33.2)
Moderate physical activity*				
	No	190 (44.2)	240 (55.8)	430 (42.4)
	Yes	326 (55.9)	257 (44.1)	583 (57.6)
Pedal or bicycle for 10 minutes daily*				
	No	498 (67.1)	244 (32.9)	742 (66.5)
	Yes	23 (6.1)	351 (93.9)	374 (33.5)
**Sitting in hours per day**				
	≤ 3 days	296 (45.7)	352 (54.3)	648 (64.9)
	> 3 days	145 (41.3)	206 (58.7)	351 (35.1)

*Note*.

* marks a significant difference between males and females *p* < 0.05

**Table 3:** Bivariate analysis of bio demographic and socio-economic factors and hypertension among those surveyed using the WHO STEPS in Jalalabad, Nangarhar, Afghanistan

**Variables**	**Categories**	**Hypertensive**	**Normotensive**	**Odds Ratio**	**95% CI**
**Age**					
	25 – 34	72 (16.0)	379 (84.0)	1	Reference
	35 – 44	85 (30.1)	197 (69.9)	2.28	1.59 – 3.26
	45 – 54	97 (46.0)	114 (54.0)	4.46	3.08 – 6.46
	55 and over	62 (43.1)	82 (56.9)	4	2.64 – 6.06
**Sex**					
	Female	207 (35.7)	373 (64.3)	1	Reference
	Male	128 (21.3)	472 (78.7)	2.05	1.58 – 2.65
**Level of education**					
	Illiterate	237 (30.3)	546 (69.7)	1	Reference
	Literate	94 (24.5)	289 (75.5)	1.33	1.01 – 1.76
**Monthly income (Afghanis)**					
	≤ 10,000	192 (26.1)	544 (73.9)	1	Reference
	> 10,000	34 (44.7)	42 (55.3)	0.44	0.27 – 0.70
**Smoking**					
	No	289 (27.7)	754 (72.3)	1	Reference
	Yes	19 (21.1)	71 (78.9)	1.43	0.85 – 2.42
**Fruit servings consumed in days per week**					
	≤ 3 days	214 (28.0)	551 (72.0)	1	Reference
	> 3 days	88 (27.2)	236 (72.8)	1.04	0.78 – 1.40
**Vegetable servings consumed days per week**					
	≤ 3 days	67 (29.1)	163 (70.9)	1	Reference
	> 3 days	264 (28.2)	671 (71.8)	1.04	0.76 – 1.44
**Type of kitchen oil**					
	Liquid	156 (33.3)	313 (66.7)	1	Reference
	Solid	156 (24.2)	489 (75.8)	1.76	1.31 – 2.37
**Vigorous physical activity**					
	No	231 (30.8)	520 (69.2)	1	Reference
	Yes	75 (20.1)	298 (79.9)	1.04	0.78 – 1.40
**Moderate physical activity**					
	No	145 (33.7)	285 (66.3)	1	Reference
	Yes	133 (22.8)	450 (77.2)	1.72	1.30 – 2.27
**Sitting in hours per day**					
	≤ 3 hours	164 (25.3)	483 (74.7)	1	Reference
	> 3 hours	113 (32.1)	239 (67.9)	0.72	0.54 – 0.95

**Table 4:** Bivariate analysis of pathophysiologic factors and hypertension among those surveyed using the WHO STEPS in Jalalabad, Nangarhar, Afghanistan

**Variables**	**Categories**	**Hypertensive**	**Normal**	**Odds Ratio**	**95% CI**
**Basic mass index**					
	Underweight	11 (14.3)	66 (85.7)	1	Reference
	Normal weight	74 (18.9)	317 (81.1)	1.4	0.70 – 2.79
	Overweight	105 (29.6)	250 (70.4)	2.52	1.28 – 4.98
	Obese	119 (43.1)	157 (56.9)	4.55	2.30 – 8.99
**Central obesity**					
	No	86 (19.1)	365 (80.9)	1	Reference
	Yes	214 (35.7)	385 (64.3)	0.42	0.32 – 0.57
**Diabetes mellitus**					
	Diabetic	61 (45.5)	73 (54.5)	1	Reference
	No diabetic	270 (26.4)	752 (73.6)	2.32	1.61 – 3.36
**Total cholesterol**					
	<190 mg/dL	142 (26.1)	402 (73.9)	1	Reference
	≥190 mg/dL	189 (30.9)	422 (69.1)	0.8	0.61 – 1.02
**Low density lipoprotein (LDL)**					
	<100 mg/dL	87 (30.9)	195 (69.1)	1	Reference
	≥100 mg/dL	244 (27.9)	629 (72.1)	1.15	0.86 – 1.54
**High density lipoprotein (HDL) borderline 40 mg/dL for male and 50 mg/dL for female**					
	<40 and 50mg/dL	264 (28.5)	661 (71.5)	1	Reference
	≥40 and 50mg/dL	70 (27.6)	184 (72.40)	1.05	0.77 – 1.43
**Triglycerides**					
	<150 mg/dL	95 (26.2)	267 (73.8)	1	Reference
	≥150 mg/dL	236 (29.8)	557 (70.2)	0.84	0.63 – 1.11

**Table 5:** Multivariable analysis of risk factors and hypertension among those surveyed using the WHO STEPS in Jalalabad, Nangarhar, Afghanistan

**Variables**	**Categories**	**Adjusted Odds Ratio**	**95% CI**	***p*-value**
**Age**				
	≤ 40 years	1	Reference	-
	> 40 years	3.42	2.50 – 4.76	<0.01
**Sex**				
	Female	1	Reference	-
	Male	0.58	0.38 – 0.88	<0.05
**Job nature**				
	Non-physical	1	Reference	-
	Physical	0.55	0.36 – 0.85	<0.01
**Central obesity**				
	BMI < 30	1	Reference	-
	BMI ≥ 30	2.1	1.49 – 2.94	< 0.01
**Diabetes mellitus**				
	No	1	Reference	-
	Yes	1.75	1.10 – 2.79	< 0.05
**Physical activity**				
	No	1	Reference	-
	Yes	0.69	0.47 – 0.99	< 0.05
**Vegetable servings consumed in days per week**				
	≤ 3 days	1	Reference	-
	> 3 days	0.59	0.38 – 0.93	< 0.05
